# Assessing the diagnostic value of qPCR for *Trichuris trichiura*: sub-analysis of a multi-country clinical trial to determine the efficacy of albendazole compared to an albendazole-ivermectin fixed dose combination

**DOI:** 10.3389/fpara.2025.1679294

**Published:** 2025-11-06

**Authors:** Pedro E. Fleitas, Michel Bengtson, Augusto Messa, Brian Bartilol, Woyneshet Gelaye, Stella Kepha, Javier Gandasegui, Áuria de Jesus, Valdemiro Novela, Inácio Mandomando, Charles Mwandawiro, Wendemagegn Enbiale, Alejandro Krolewiecki, Jose Muñoz, Martin Rono, Lisette van Lieshout

**Affiliations:** 1 Barcelona Institute for Global Health – ISGLOBAL, Barcelona, Spain; 2 Parasitology Research Group, Leiden University Center for Infectious Diseases (LUCID), Leiden University Medical Center (LUMC), Leiden, Netherlands; 3 Centro de Investigaçao em Saúde da Manhiça (CISM), Maputo, Mozambique; 4 Facultat de Medicina i Ciències de la Salut, Universitat de Barcelona (UB), Barcelona, Spain; 5 Eastern and Southern Africa Centre of International Parasite Control, Kenya Medical Research Institute, Nairobi, Kenya; 6 College of Medicine and Health Sciences, Bahir Dar University, Bahir Dar, Ethiopia; 7 Wellcome Sanger Institute, Hinxton, Cambridgeshire, United Kingdom; 8 Instituto Nacional de Saúde (INS), Maputo, Mozambique; 9 Global Health and Tropical Medicine (GHTM), Instituto de Higiene e Medicina Tropical (IHMT), Universidade NOVA de Lisboa, Lisboa, Portugal; 10 Instituto de Investigaciones de Enfermedades Tropicales/CONICET, Universidad Nacional de Salta, Orán, Argentina; 11 Fundación Mundo Sano, Buenos Aires, Argentina; 12 International Health Department, Hospital Clínic de Barcelona, Barcelona, Spain; 13 Centre for Geographic Medicine Research (Coast), Kenya Medical Research Institute-Wellcome Trust Research Programme, Kilifi, Kenya

**Keywords:** diagnostics, qPCR, Kato-Katz, *Trichuris trichiura*, albendazole ivermectin fixed-dose efficacy

## Abstract

*Trichuris trichiura* remains a major global public health concern, particularly in low-resource settings where standard anthelmintic regimens are limited. This study evaluated the diagnostic performance of real-time PCR (qPCR) compared to the Kato-Katz (KK) method in assessing the efficacy of a fixed-dose combination (FDC) of albendazole and ivermectin versus albendazole for the treatment of *T. trichiura*. The study was embedded within the ALIVE clinical trial (NCT05124691), a phase 2/3 trial conducted in Kenya, Mozambique, and Ethiopia. Stool samples were collected at baseline and 21 ± 7 days post-treatment, with KK performed on fresh samples and qPCR on ethanol-preserved aliquots. In total 534 participants were selected based on positive KK and qPCR at baseline and complete data post-treatment. The primary endpoint was cure rate (CR) by KK and qPCR; secondary endpoints included egg reduction rate (ERR) and cycle threshold (Ct) value incrementation rate (CtIR). Additionally, machine learning algorithms were used to predict infection intensity from qPCR Ct-values and demographic variables. qPCR confirmed the superior efficacy of FDC compared to albendazole as previously shown by KK, but discrepancies were observed in CRs between qPCR and KK, particularly lower qPCR CRs for FDC×1 and FDC×3. Concordance between stool egg counts and Ct-value decreased post-treatment, likely due to reduced KK sensitivity in low-intensity infections. ERR and CtIR showed parallel patterns of efficacy across treatment arms. Machine learning models showed good performance for predicting baseline infection intensity. While not interchangeable, qPCR complements KK and enhances the precision of drug efficacy evaluation in helminth clinical trials.

## 1 Introduction

*Trichuris trichiura* is a soil-transmitted helminth (STH) that affects an estimated 460 million individuals worldwide, particularly in tropical and subtropical regions with inadequate sanitation (Pullan et al., 2014). Transmission occurs through the ingestion of embryonated eggs in contaminated soil or food (Jourdan et al., 2018). Chronic infections cause significant morbidity, including anemia, diarrhea, and cognitive and physical impairments, especially in children (Jourdan et al., 2018).

The current standard for controlling *T. trichiura* infections involves mass drug administration (MDA) using benzimidazole derivatives such as albendazole or mebendazole. While these drugs are effective against other STHs like *Ascaris lumbricoides* and hookworms, their efficacy against *T. trichiura* is suboptimal, with cure rates as low as 40% (Moser et al., 2017). This limitation necessitates exploration of alternative treatment strategies, such as combination therapies. Fixed-dose combination tablets (FDC) of albendazole and ivermectin have emerged as promising candidates, demonstrating superior efficacy to single-dose albendazole against *T. trichiura* (Krolewiecki et al., 2025). However, robust efficacy evaluations using sensitive diagnostic tools are essential to determine true drug efficacy. Evaluating antihelmintic efficacy requires diagnostic methods that reliably detect changes in parasite burden pre- and post-treatment. The World Health Organization recommends the Kato-Katz method (KK) for detecting STH eggs in stool samples due to its ability to quantify eggs per gram (EPG) of stool, measure infection intensity, simplicity, low cost and field applicability (Chammartin et al., 2013). Nevertheless, KK has significant limitations in low-intensity infections, including post-treatment scenarios. Its sensitivity decreases with diminishing egg counts, leading to potential overestimation of efficacy in clinical trials (Nikolay et al., 2014).

Real-time polymerase chain reaction (qPCR) provides a more sensitive alternative to KK, especially in low-prevalence areas or low-intensity infections, by detecting helminth DNA in stool (Verweij and Stensvold, 2014; Cools et al., 2019). qPCR can also differentiate between helminth species with morphologically similar eggs (e.g. hookworms) and significantly improves the detection of *Strongyloides stercoralis* compared to conventional microscopic methods, which are limited by low sensitivity, and labor-intensive methods like the Baermann technique (Becker et al., 2015). However, interpreting qPCR results to assess infection intensity requires careful consideration. The quantity of parasite DNA detected does not directly correspond to egg counts due to biological variables (e.g., egg maturation and developmental stage) that can lead to variation in genomic DNA copy number per egg (Papaiakovou et al., 2019; Cools et al., 2021). Additionally, some qPCR targets may be present in multiple copies or as tandem repeats in the genome, further complicating quantitative interpretation. Consequently, while lower cycle threshold (Ct) values generally correlate with higher parasite loads, the relationship between Ct-values and true infection intensity remains complex and incompletely characterized (Papaiakovou et al., 2019; Cools et al., 2021).

qPCR serves as a powerful complement to KK for assessing drug efficacy, and their combined use provides a more comprehensive and accurate evaluation of anthelmintic treatment efficacy. In this study, we aimed to assess the diagnostic value of qPCR relative to KK within a clinical trial evaluating the efficacy of FDC compared to albendazole monotherapy for the treatment of *T. trichiura*. Additionally, we explored the potential of using machine learning algorithms to predict infection intensity based on qPCR results, addressing the current challenges in translating qPCR output into standardized intensity measures and advancing the utility of molecular diagnostics in helminth control programs.

## 2 Materials and methods

### 2.1 Study design

This study corresponds to Exploratory Objective 2 of the ALIVE clinical trial, an adaptive, phase 2/3, single-blinded (outcome assessor–blinded), randomized, multicenter, parallel-group, active-controlled, superiority trial. This objective focuses on evaluating the detection of parasite-specific DNA using qPCR to assess the primary efficacy outcome and compare it with conventional stool microscopy (Krolewiecki et al., 2022). The main objectives and primary findings of the clinical trial have already been published (Krolewiecki et al., 2025). The trial evaluated the safety and efficacy of an orodispersible fixed-dose co-formulation (FDC) of albendazole (400 mg) and ivermectin (9 mg or 18 mg) for the treatment of *T. trichiura*, hookworms, and *S. stercoralis*. The FDC was administered as either a single dose (FDC×1) or three consecutive daily doses (FDC×3) and compared with a single dose of albendazole (400 mg) as the control group, reflecting standard public health practices (Krolewiecki et al., 2022). Recruitment was conducted in schools across Kwale County (Kenya), Bahir Dar Zuria (Ethiopia), and Manhiça District (Mozambique).

To measure the efficacy of the treatment arms, stool samples were collected at baseline and 21 ± 7 days after treatment. Fresh samples were analyzed in duplicate using the KK method, while qPCR analysis was performed on ethanol-preserved samples. This study only includes the analysis of the diagnosis by KK thick smears and qPCR for *T. trichiura* infection. A summary of the qPCR findings for *A. duodenale, A. lumbricoides*, *S. stercoralis* and *Schistosoma* spp. is presented in **Supplementary Tables S1, S2** of the **Supplementary Material**.

Ethical approvals were obtained from the respective national and institutional ethics committee in each participating country. The trial was registered at ClinicalTrials.gov (NCT05124691). Parents or legal guardians of participating children provided written informed consent, while participants aged 12 years or older also provided written assent.

### 2.2 Laboratory procedures

One aliquot of ethanol-preserved samples (baseline and post-treatment) from all three ALIVE trial sites was shipped to the KEMRI-Wellcome Trust Laboratories in Kilifi, Kenya where all qPCR-related experiments were conducted.

#### 2.2.1 Nucleic acid extraction

Genomic DNA was extracted from ALIVE study stool samples in batches of 24, each including a negative extraction control consisting of phosphate-buffered saline (PBS; Sigma-Aldrich, cat. D8537). Extractions were performed using the QIAamp DNA Mini Kit (Qiagen, Hilden, Germany; cat. 51306), following the manufacturer’s protocol with minor modifications to enhance inhibitor removal. Briefly, 250 µL of ethanol-preserved stool suspension was transferred into 2 mL PowerBead tubes (Qiagen, cat. 13113050) containing 1.4 mm ceramic beads. Tubes were centrifuged at 14,000 × g for 1 minute, and the ethanol supernatant was discarded. The resulting pellet was washed with 1,000 µL of PBS, centrifuged again, and the supernatant removed. To reduce PCR inhibitors, 200 µL of 2% polyvinylpolypyrrolidone (PVPP; Supelco, cat. 77627) was added to each tube. Samples were then subjected to bead-beating for 10 minutes using the TissueLyser II (Qiagen, Hilden, Germany), followed by freezing at –80°C for 30 minutes. Tubes were returned to room temperature, vortexed briefly, and incubated at 100°C for 10 minutes. After a quick centrifugation, DNA extraction proceeded according to the QIAamp DNA Mini Kit protocol, except that 400 µL of AL buffer spiked with Phocine Herpesvirus-1 (PhHV; EVAg, cat. 011V-00884) was used as an internal control for extraction and to assess amplification efficiency. A final elution volume of 200 µL was obtained, and DNA was stored at 2–8°C until qPCR analysis, which was conducted within the same calendar year (2023).

#### 2.2.2 Real-time PCR

DNA samples from the ALIVE trial were analyzed in duplicate using two multiplex qPCR panels: the ST panel targeting *Schistosoma* spp. and *T. trichiura* and the ANAS panel targeting *Ancylostoma duodenale*, *Necator americanus*, *A. lumbricoides*, and *S. stercoralis*. The *N. americanus* qPCR was excluded from further analysis due to technical challenges during the ALIVE study period, likely due to the design of two probes for *N. americanus* and potential incompatibility issues between the fluorophores of the probes and the real-time PCR system used in this study. Briefly, two *N. americanus* probes designed as BHQ™ probes were non-functional in the multiplex design and further optimization is required for this target i.e. the *N. americanus* probes can be procured as minor groove binding (MGB) detection probes that use a specific molecule combined with a 3’quencher to increase the melting temperature of the probes which is required to bind before the primers bind.

Both multiplex qPCR included primers and probes for Phocine Herpesvirus-1 (PhHV) as an internal control to monitor extraction and amplification efficiency. The ST multiplex panel was performed in both Phase II and Phase III of the ALIVE study, whereas the ANAS multiplex panel was conducted exclusively in Phase III. This design reflects the study’s objectives: treatment efficacy against *T. trichiura* was evaluated in both phases, while efficacy against hookworms and *S. stercoralis* was assessed only in Phase III (Krolewiecki et al., 2022).

The oligonucleotide sequences and concentrations for soil-transmitted helminth detection and the internal control have been previously described (Kaisar et al., 2017). qPCR reactions were performed on a QuantStudio™ 5 real-time PCR System and an Applied Biosystems 7500 real-time PCR System (Applied Biosystems, Foster City, CA, USA). A sample was considered positive for a given target if both replicates showed amplification curves with Ct-values ≤35. Samples were classified as negative when both replicates had Ct-values >35. Reactions were repeated in cases of discordant results between replicates or if the Ct difference between replicates exceeded 3.3 cycles. For all samples with a negative qPCR result, the Ct-value was recorded as 36.

### 2.3 Statistical analysis

The study population consisted of a per-protocol population from the ALIVE clinical trial, defined as participants who were randomized, not withdrawn, not lost to follow-up, and confirmed to be infected with *T. trichiura* at baseline by both KK and qPCR.

The association between EPG obtained by the KK and the Ct-values from qPCR was assessed using Spearman’s rank correlation coefficient. This analysis was conducted separately for the baseline population and the post-treatment population, including only participants who tested positive by KK or qPCR.

The concordance between KK and qPCR results was assessed using Cohen’s kappa coefficient.

The primary efficacy outcome was the cure rate (CR), defined as the percentage of participants cured 21 ± 7 days post-treatment. For the KK thick smear method, cure was determined by the absence of *T. trichiura* eggs in stool samples collected 21 ± 7 days after treatment among participants who were positive at baseline. For qPCR, cure was defined as a Ct-value equal to 36 at 21 days post-treatment in participants with confirmed infection at baseline. The cure rate was calculated for both KK and qPCR, along with the corresponding 95% confidence intervals (CIs), assuming a binomial distribution. Comparisons of cure rates between treatment arms for each diagnostic method were conducted using the Cochran–Mantel–Haenszel test, accounting for the effect of study sites. Fisher’s exact test was used to compare the cure rates obtained by KK and qPCR.

As a secondary efficacy measure, the egg reduction rate (ERR) was calculated to assess the decrease in *T. trichiura* egg burden following treatment. The ERR was determined by the following formula, using the arithmetic mean of EPG:


$$ERR=1-\frac{Mean\;of\;egg\;count\;posttreatment}{Mean\;of\;egg\;count\;at\;baseline}$$

ditionally, as an exploratory efficacy measure, the mean Ct-value incrementation rate (CtIR), analogous to the ERR, was calculated to assess changes in Ct-values. The CtIR was determined by the following formula, using the arithmetic mean of Ct-values:


$$CtIR=1-\frac{36-mean\;of\;Ct\;values\;posttreatment}{36-mean\;of\;Ct\;values\;at\;baseline}$$

th the ERR and CtIR, along with their corresponding 95% confidence intervals for the means, were estimated using bootstrap resampling techniques.

To compare differences in ERR and CtIR between treatment groups, we employed an ANCOVA. For ERR, the logarithm of egg counts post-treatment was used as the dependent variable, with site and treatment included as fixed factors, and the logarithm of baseline egg counts as a covariate. Similarly, for CtIR, the post-treatment Ct-values served as the dependent variable, with site and treatment as fixed factors, and baseline Ct-values as a covariate. All descriptive and statistical analyses were performed with R software version 4.1.2 (R Core Team, 2013).

### 2.4 Machine learning

We assessed the potential of machine learning techniques to predict infection intensity using qPCR Ct-values in combination with demographic variables, such as age and body mass index (BMI). The incorporation of the demographic variables was done to improve the predictive ability of the machine learning models, as these variables are known to be associated with infection intensity.

Baseline data from study participants were utilized, with infection intensity classified as mild (EPG < 1000) or moderate to high (EPG ≥ 1000). The dataset was split into training (70%) and testing (30%) subsets. Ten machine learning models were evaluated: Logistic Regression, Support Vector Machine (SVM), K-Nearest Neighbors (KNN), Decision Tree, Random Forest, Gradient Boosting, AdaBoost, XGBoost, Naive Bayes, and Neural Networks. Stratified 10-fold cross-validation was used to assess model performance, with metrics including the area under the receiver operating characteristic curve (AUC-ROC). The model achieving the best cross-validation performance underwent hyperparameter tuning to optimize predictive accuracy. Following optimization, the selected model was applied to the test set, where its predictive performance was evaluated using AUC-ROC, sensitivity, and specificity for infection intensity classification. In addition, the model was applied to predict the intensity of post-treatment infection. All machine learning analyses were conducted using Python, leveraging libraries such as Scikit-learn, and pandas for model development and evaluation.

## 3 Results

### 3.1 *T. trichiura* per-protocol population

**Figure 1:**
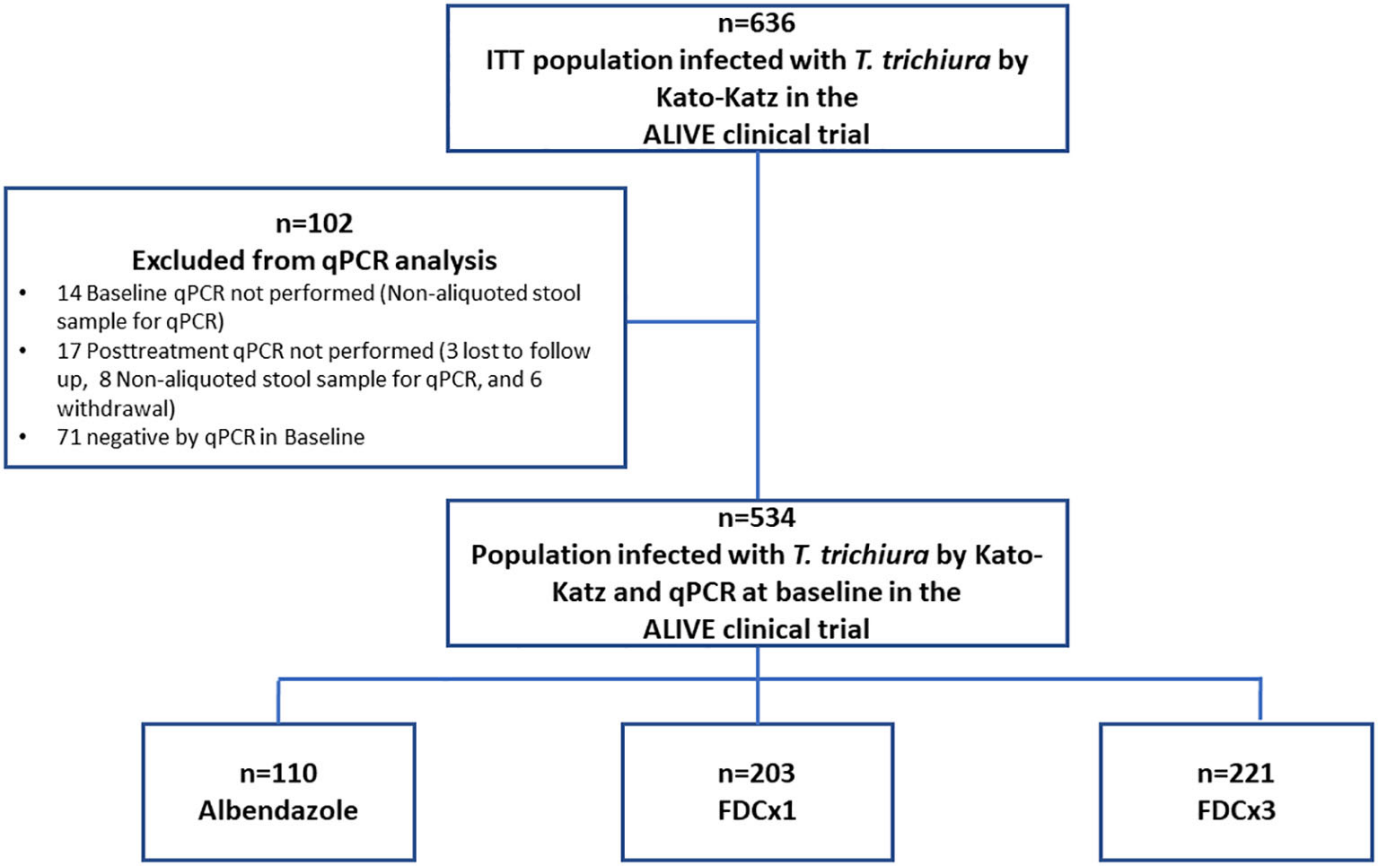
Flowchart of the per-protocol population.

The per-protocol population consisted of 534 participants, representing 84% of the intention-to-treat population from the ALIVE clinical trial (**Figure 1**). The primary reason for exclusion was a negative qPCR result at baseline.

The baseline characteristics are shown in **Supplementary Table S3**. The majority of participants were from Kenya (75%) and Mozambique (25%), with only a single case reported in Ethiopia. Furthermore, 90% of the infections were classified as mild in intensity.

### 3.2 Efficacy of albendazole and FDC for *T. trichiura* infection measured by KK and qPCR

At baseline (n=534), a moderate inverse correlation was observed between EPG and Ct-values (Spearman’s ρ = –0.48; p < 0.001), consistent with the expected relationship whereby higher egg counts correspond to lower Ct-values. In contrast, among participants who remained positive by either KK or qPCR following treatment (n = 188), a weak positive correlation emerged (Spearman’s ρ = 0.18; p = 0.013), indicating a disruption of the baseline pattern. This is because the agreement between the two diagnostic methods in this group was poor, with a negative kappa coefficient (κ = –0.487; p < 0.001), suggesting systematic disagreement and a tendency toward divergent classification of infection status post-treatment.

Agreement between qPCR and KK for assessing post-treatment infection status also varied across treatment arms. In the FDC×1 group, agreement was classified as fair (κ = 0.229, p = 0.003). In contrast, the albendazole group showed only slight agreement (κ = 0.127, p = 0.159), and the FDC×3 group demonstrated poor agreement (κ = 0.050, p = 0.300). However, in both of these arms, the observed agreement was not statistically different from what would be expected by chance.

**Figure 2:**
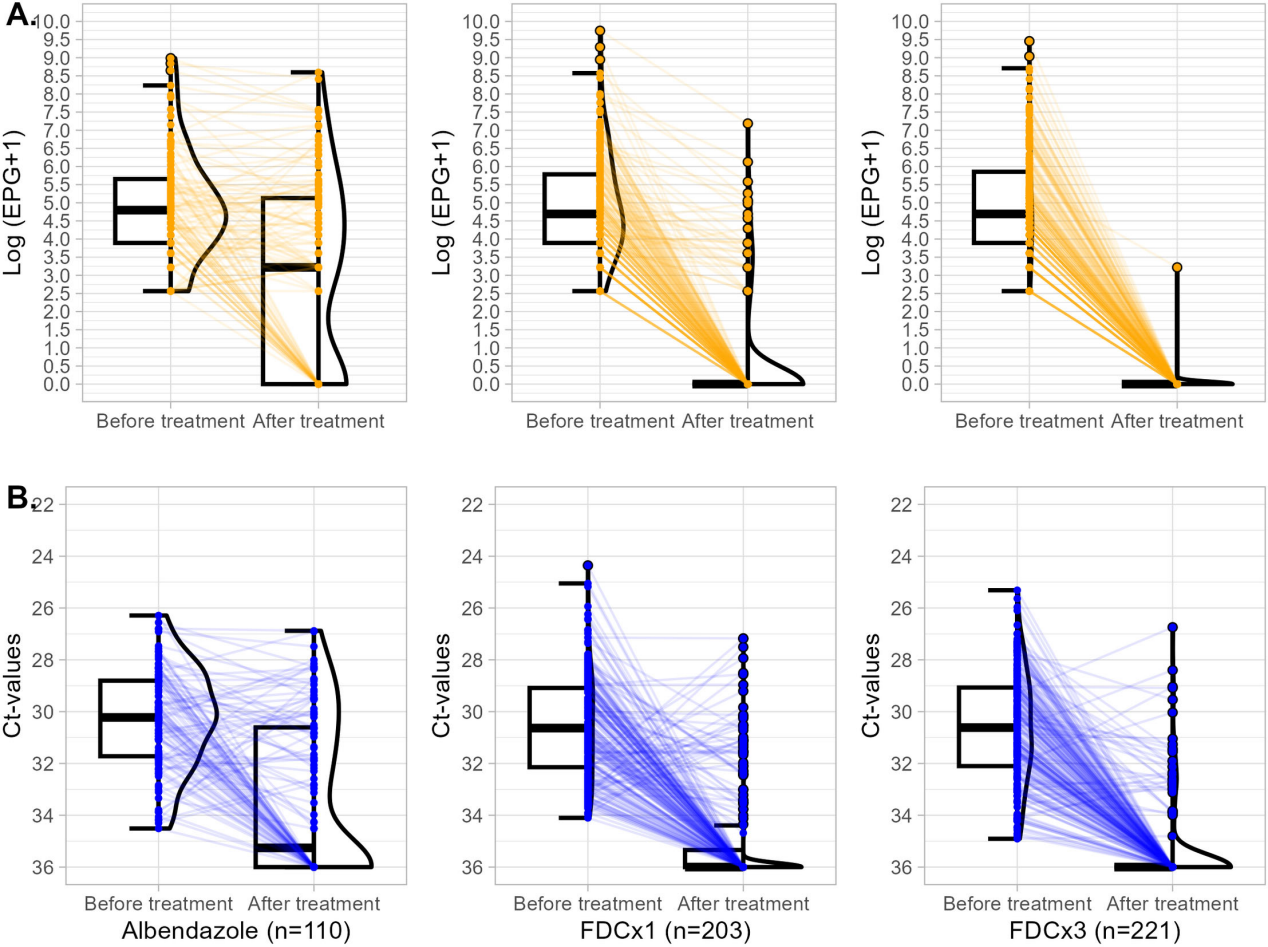
Box-violin plots illustrating the variation in EPG and Ct-values before and after treatment. **(A)** Variation in log (EPG + 1) values. **(B)** Variation in Ct values. Treatment arms are displayed from left to right: albendazole, FDC×1, and FDC×3.

The efficacy results are summarized in **Table 1**, while variations in EPG and Ct-values between baseline and post-treatment for each treatment arm are illustrated in **Figure 2**. Cure rates measured by both KK and qPCR were significantly higher for FDC×1 (83.7% by KK, 74.9% by qPCR) and FDC×3 (99.5% by KK, 85.1% by qPCR) compared to albendazole (35.5% by KK, 50.0% by qPCR) (p < 0.001 for all comparisons within the same diagnostic method). Additionally, FDC×3 demonstrated a higher cure rate compared to FDC×1, as measured by both KK and qPCR (p < 0.001 for both comparisons). However, discrepancies were observed between cure rates measured by qPCR and KK within the same treatment arms. In the albendazole group, qPCR yielded higher cure rates compared to KK (p = 0.041). Conversely, for both FDC×1 (p = 0.037) and FDC×3 (p < 0.001), qPCR reported lower cure rates than those measured by KK.

**Table 1:** Efficacy result by KK and qPCR.

Efficacy outcome	ALB (n=110)	FDCx1 (n=203)	FDCx3 (n=221)
Cured by Kato-Kats (n)	39	170	220
Cured by qPCR (n)	55	152	188
Cure rate by KK (95%CI)	35.5 (26.7, 45.2)	83.7 (77.8, 88.4)	99.5 (97.1, 99.9)
Cure rate by qPCR (95%CI)	50.0 (40.8, 59.2)	74.9 (68.2, 80.6)	85.1 (79.5, 89.4)
Baseline median EPG (IQR)	120 (48, 285)	108 (48, 324)	108(48, 348)
Baseline median Ct-values (IQR)	30.2 (28.8, 31.7)	30.6 (29.1, 32.2)	30.6 (29.1, 32.1)
Post-treatment median EPG (IQR)	24 (0, 168)	0 (0, 0)	0 (0, 0)
Post-treatment median Ct-values (IQR)	35.0 (30.6, 50.0)	50.0 (35.0, 50.0)	50.0 (50.0, 50.0)
Arithmetic mean ERR	42.9 (2.5, 59.2)	96.4 (94.3, 98.0)	99.9 (99.9, 100.0)
Arithmetic mean CtIR	53.0 (42.5, 63.3)	79.3 (73.0, 84.5)	88.8 (84.0, 92.2)

Consistent with cure rate patterns, ERRs demonstrated significantly greater efficacy for both FDC×1 (96.4%) and FDC×3 (99.9%) compared to albendazole (42.9%) (p < 0.001 for both comparisons). Similarly, CtIRs mirrored these findings, with higher values observed for FDC×1 (79.3%) and FDC×3 (88.8%) relative to albendazole (53.0%) (p < 0.001 for both comparisons). These parallel trends between ERRs and CtIRs support the robustness of the efficacy estimates across KK and qPCR.

### 3.3 Machine learning modeling to identify *T. trichiura* infection intensity

Among the ten models evaluated, logistic regression achieved the highest performance in stratified 10-fold cross-validation, with a mean AUC-ROC of 0.84 (IC 95%: 0.77, 0.92) (**Supplementary Figure S1, Supplementary Material**). The optimized model demonstrated an AUC-ROC of 0.76 on the test set, along with a sensitivity, for detecting moderate or heavy infections, of 0.83 (IC 95%: 0.71, 0.90) and a specificity of 0.66 (IC 95%: 0.62, 0.69).

**Figure 3:**
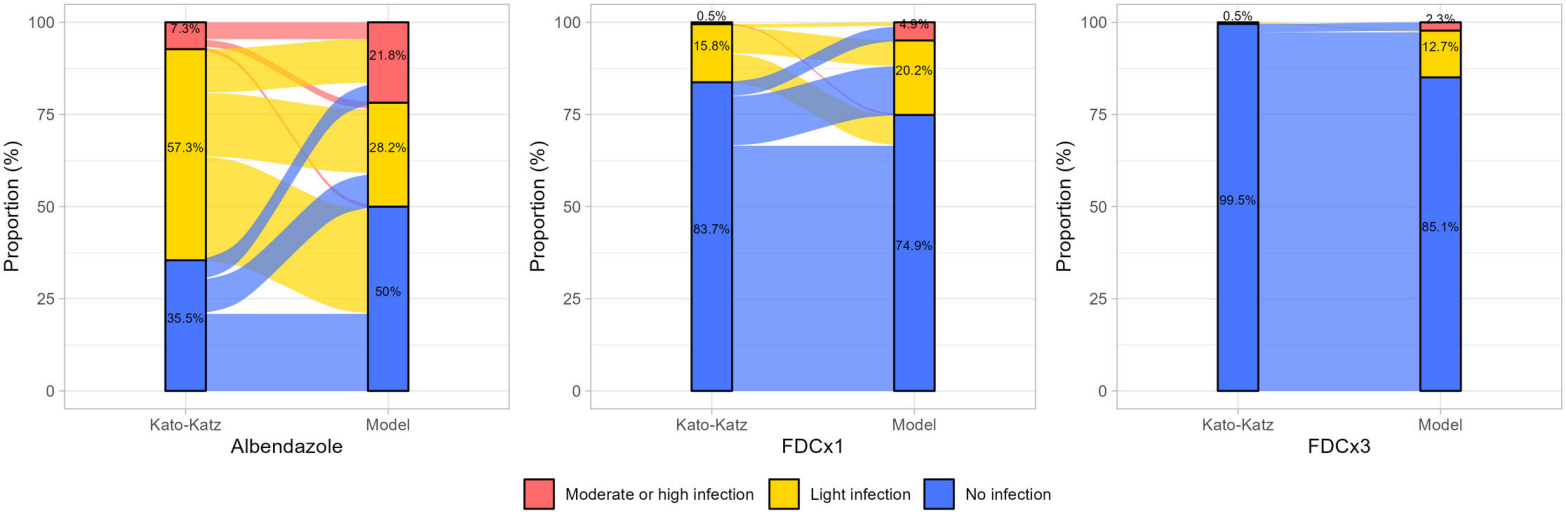
Comparison of post-treatment infection intensity classifications between Kato-Katz and the predictive model across treatment arms. The bars represent the proportion of participants classified as having no infection (blue), light infection (yellow), and moderate-to-high infection (red) intensity by each method. The Sankey-style flow visualization depicts reclassification trends, highlighting discrepancies between KK and the model.

Comparison between the model and KK for post-treatment infection classification revealed that the model identified a higher proportion of moderate-to-high intensity infections across all treatment arms. Specifically, the model classified 21.8% of albendazole-treated participants, 4.9% of those receiving FDC×1, and 2.3% of the FDC×3 group as having moderate-to-high infections after treatment, compared to 7.3%, 0.5%, and 0.5%, respectively, using KK (**Figure 3**).

## 4 Discussion

This study highlights important discrepancies between KK and qPCR in evaluating the efficacy of albendazole and FDC therapy against *T. trichiura*. While both methods confirmed the superior efficacy of FDC compared to albendazole, qPCR yielded lower cure rates for FDCx1 and FDCx3 but a higher cure rate for albendazole. Although qPCR exhibited near-perfect agreement with KK at baseline (**Supplementary Table S1, Supplementary Material**), post-treatment agreement diminished to fair, resulting in poor agreement among participants classified as cured in the per-protocol population. These differences extended to infection intensity classification, with the model identifying a higher proportion of moderate/high-infection intensity post-treatment compared to KK. Collectively, these findings highlight fundamental differences in how each diagnostic tool captures treatment outcomes and underscore important considerations for interpreting drug efficacy results in clinical trials.

For *T. trichiura* infections, anthelmintic efficacy estimates vary substantially across study sites and diagnostic methods (Vlaminck et al., 2019). While some studies report lower efficacy with qPCR versus KK, others have found similar or even higher cure rates using molecular diagnostics (Vlaminck et al., 2019; Keller et al., 2020; Matamoros et al., 2024). These discrepancies may reflect variations in local infection intensity, methodological differences, and inherent differences in diagnostic sensitivity and specificity between the two methods. Nevertheless, in our study, the majority of participants infected with *T. trichiura* were from Kenya, and the efficacy results for albendazole aligned with prior regional findings (Kepha et al., 2024).

A critical consideration for qPCR-based efficacy assessment is the potential persistence of non-viable parasite DNA post-treatment, which could lead to false-positive results. In our study, efficacy was assessed between 14 and 28 days post-treatment, aligning with the WHO guidelines for evaluating anthelmintic drug efficacy against soil-transmitted helminths (Levecke et al., 2014). Recent evidence suggests that efficacy should be evaluated between 18 and 24 days post-treatment, although the WHO recommended window still leads to adequate efficacy results (Welsche et al., 2024). Therefore, the positive qPCR results observed at 21 ± 7 days post-treatment in our study are unlikely to be due to residual DNA from non-viable parasites, supporting qPCR reliability in assessing treatment efficacy within this timeframe.

Several studies have explored the use of qPCR-derived Ct-values or genome equivalents per ml of stool DNA extract to quantify the intensity of STH infections (Kaisar et al., 2017; Cools et al., 2019; Barda et al., 2020). While some correlation has been found between molecular measures and egg counts by microscopy, there is no consensus on translating molecular measures into microscopy defined intensity categories (Papaiakovou et al., 2019; Cools et al., 2021). We applied machine learning to improve qPCR-based intensity predictions. Although the model demonstrated good predictive performance at baseline among participants who were positive by both KK and qPCR, its post-treatment performance was less consistent, primarily due to the low agreement observed between qPCR and KK classifications after treatment. The decline in agreement is likely driven by the decreased sensitivity of KK in detecting low-intensity infections, which become more common following treatment. As a result, KK may fail to identify cases that are still detectable by qPCR, leading to fewer microscopy-positive cases and diminished agreement between the two diagnostic methods. Machine-learning models can serve as a valuable complementary tool for estimating infection intensity in moderate- and high-burden settings, integrating features such as Ct values and other available variables, without relying on KK. However, their utility is limited in low-intensity infections, where the weak correlation between KK egg counts and qPCR signals diminishes model accuracy and interpretability. In our view, there is a clear need to establish dedicated metrics for qPCR-based assessment of STH infection intensity that are independent of traditional microscopy-based standards.

Although the cure rate is widely used as a measure of efficacy in clinical trials, its application is debated due to dependence on baseline infection intensity and the sensitivity of the diagnostic method employed (Montresor, 2011). Thus, WHO recommends ERR as the primary efficacy metric (World Health Organization, 2013). However, qPCR-based ERR faces similar challenges to intensity quantification, as DNA quantification cannot be directly translated into egg counts. To address this, we used ΔCt-values as an egg-reduction proxy, allowing us to assess differences in drug efficacy across treatment arms. The CtIR calculated with qPCR, analogous to the ERR calculated via KK, demonstrated the same significant differences between treatment groups and supported similar conclusions regarding efficacy. Thus, using CtIR provides a straightforward and practical method to evaluate reductions in parasite load without the need for complex DNA-to-egg conversions. Moreover, it is important to recognize that no parasitological method has produced ERR results identical to KK (Vlaminck et al., 2019), nevertheless, alternative diagnostic techniques, including qPCR, have shown substantial agreement in assessing drug efficacy. Therefore, while qPCR may not replicate KK’s quantitative outputs, its ability to support comparable efficacy conclusions underscores its value as a complementary diagnostic tool in the evaluation of anthelmintic treatments.

One limitation of this study is that efficacy results were only evaluated in participants who tested positive on both the Kato-Katz test and qPCR at baseline. As a result, individuals with discordant results at baseline were excluded from the efficacy analysis, which may limit the generalizability of the findings to this population. Also, our study was limited by the fact that only one stool sample was used at each time point, and qPCR results may vary between samples (Keller et al., 2020), suggesting that multiple samples per individual could provide more reliable data. Although hookworms were a secondary endpoint in the ALIVE trial for efficacy assessment, qPCR optimization was only successful for *A. duodenale* and not for *N. americanus*, due to the use of probes with incompatible chemistry relative to the original assay design. As a result, we were unable to evaluate treatment efficacy for hookworm infections using qPCR.

## Data Availability

The datasets presented in this study can be found in online repositories. The names of the repository/repositories and accession number(s) can be found below: Infectious Diseases Data Observatory, https://www.iddo.org/.
