# Supplementary material for: Assessing the diagnostic value of qPCR for Trichuris trichiura: sub-analysis of a multi-country clinical trial to determine the efficacy of albendazole compared to an albendazole-ivermectin fixed dose combination
Source: Front Parasitol. 2025 Nov 6;4:1679294. doi: 10.3389/fpara.2025.1679294 (PMC12629931; doi:10.3389/fpara.2025.1679294)

Supplementary Material

# Table S1: Summary of ST multiplex qPCR results in the ALIVE clinical trial population, including participants who were randomized, completed follow-up, and had ST qPCR data (n=942).

| Site | Ethiopia | | | Kenya | | | Mozambique | | | Total | | |
| --- | --- | --- | --- | --- | --- | --- | --- | --- | --- | --- | --- | --- |
| Treatment | ALB (n=89) | FDCx1 (n=103) | FDCx3 (n=96) | ALB (n=103) | FDCx1 (n=194) | FDCx3 (n=200) | ALB (n=32) | FDCx1 (n=62) | FDCx3 (n=63) | ALB (n=224) | FDCx1 (n=359) | FDCx3 (n=359) |
| Baseline |  | | | | | | | | | | | |
| *T. trichiura* positives by KK | 0 | 1 | 0 | 89 | 174 | 182 | 31 | 61 | 63 | 120 | 236 | 245 |
| *T. trichiura* positives by qPCR | 0 | 1 | 0 | 85 | 153 | 169 | 24 | 53 | 58 | 109 | 207 | 227 |
| *Schistosoma spp* positives by qPCR | 33 | 28 | 25 | 1 | 2 | 1 | 1 | 0 | 1 | 35 | 30 | 27 |
| Posttreatment |  | | | | | | | | | | | |
| *T. trichiura* positives by KK | 0 | 1 | 0 | 65 | 34 | 3 | 12 | 2 | 0 | 77 | 37 | 3 |
| *T. trichiura* positives by qPCR | 0 | 1 | 0 | 39 | 49 | 36 | 23 | 15 | 3 | 62 | 65 | 39 |
| *Schistosoma spp* positives by qPCR | 25 | 32 | 26 | 2 | 4 | 1 | 0 | 1 | 0 | 27 | 37 | 27 |
| Cure rates (%) |  | | | | | | | | | | | |
| *T. trichiura by KK* | - | 0 | - | 27 | 80.5 | 98.4 | 61.3 | 96.7 | 100.0 | 35.8 | 84.3 | 98.8 |
| *T. trichiura by qPCR* | - | 0 | - | 54.1 | 68.0 | 78.7 | 4.2 | 71.7 | 94.8 | 43.1 | 68.6 | 82.8 |
| *Schistosoma spp by qPCR* | 24.2 | -14.3 | -4.0 | -100.0 | -100.0 | 0 | 100.0 | - | 100.0 | 22.9 | -23.3 | 0 |

Kappa Agreement between Kato-Katz and qPCR for baseline *T. trichiura* infections: 0.818 (p<0.001)

Kappa Agreement between Kato-Katz and qPCR for posttreatment *T. trichiura* infections: 0.343 (p<0.001)

# Table S2: Summary of ANAS multiplex qPCR results in the Phase III ALIVE clinical trial population, including participants who were randomized, completed follow-up, and had ANAS qPCR data (n=825).

| Site | Ethiopia | | | Kenya | | | Mozambique | | | Total | | |
| --- | --- | --- | --- | --- | --- | --- | --- | --- | --- | --- | --- | --- |
| Treatment | ALB (n=89) | FDCx1 (n=103) | FDCx3 (n=96) | ALB (n=80) | FDCx1 (n=151) | FDCx3 (n=149) | ALB (n=32) | FDCx1 (n=62) | FDCx3 (n=63) | ALB (n=201) | FDCx1 (n=316) | FDCx3 (n=308) |
| Baseline |  | | | | | | | | | | | |
| Hookworms positives by KK | 86 | 94 | 90 | 12 | 25 | 20 | 1 | 2 | 0 | 99 | 121 | 110 |
| *A. duodenale* positives by qPCR | 0 | 0 | 0 | 1 | 0 | 5 | 0 | 0 | 0 | 1 | 0 | 5 |
| *A. lumbricoides* positives by KK | 0 | 0 | 0 | 2 | 1 | 6 | 13 | 17 | 17 | 15 | 18 | 23 |
| *A. lumbricoides* positives by qPCR | 0 | 0 | 1 | 2 | 2 | 6 | 13 | 22 | 21 | 15 | 24 | 28 |
| *S. stercoralis* positives by Baermann | 11 | 19 | 17 | 4 | 17 | 18 | 0 | 0 | 0 | 15 | 36 | 35 |
| *S. stercoralis*  positives by qPCR | 1 | 4 | 4 | 1 | 8 | 7 | 0 | 0 | 1 | 2 | 12 | 12 |
| Posttreatment |  | | | | | | | | | | | |
| Hookworms positives by KK | 31 | 17 | 3 | 2 | 10 | 0 | 0 | 0 | 0 | 33 | 27 | 3 |
| *A. duodenale* positives by qPCR | 0 | 0 | 0 | 0 | 0 | 1 | 0 | 0 | 0 | 0 | 0 | 1 |
| *A. lumbricoides* positives by KK | 0 | 0 | 0 | 0 | 0 | 0 | 0 | 0 | 2 | 0 | 0 | 2 |
| *A. lumbricoides* positives by qPCR | 0 | 0 | 0 | 0 | 0 | 1 | 0 | 2 | 2 | 0 | 2 | 3 |
| *S. stercoralis* positives by Baermann | 2 | 1 | 1 | 0 | 3 | 0 | 0 | 0 | 0 | 2 | 4 | 1 |
| *S. stercoralis*  positives by qPCR | 2 | 0 | 0 | 1 | 1 | 1 | 0 | 0 | 0 | 3 | 1 | 1 |

Kappa Agreement between Kato-Katz and qPCR for baseline *A. lumbricoides* infections: 0.693 (p<0.001)

Kappa Agreement between Kato-Katz and qPCR for posttreatment *A. lumbricoides* infections: -0.003 (p=0.055)

Kappa Agreement between Baermann and qPCR for baseline *S. stercoralis* infections: 0.137 (p<0.005)

Kappa Agreement between Baermann and qPCR for posttreatment *S. stercoralis* infections: 0.160 (p=0.272)

# Table S3: Baseline characteristics of *T. trichiura*-infected participants identified by Kato-Katz and qPCR

| **Site** | **Ethiopia** | | | **Kenya** | | | **Mozambique** | | | **Total** | | |
| --- | --- | --- | --- | --- | --- | --- | --- | --- | --- | --- | --- | --- |
| **Treatment** | **ALB** | **FDCx1** | **FDCx3** | **ALB** | **FDCx1** | **FDCx3** | **ALB** | **FDCx1** | **FDCx3** | **ALB** | **FDCx1** | **FDCx3** |
| **n** | 0 | 1 | 0 | 86 | 149 | 163 | 24 | 53 | 58 | 110 | 203 | 221 |
| **Median age (IQR)** | 0(0) | 11(11, 11) | 0(0) | 11(8, 13) | 10(8, 12) | 11(8, 12) | 9(7, 12) | 8(7, 10) | 8.5(7, 10) | 10.5(8, 12) | 10(8, 12) | 10(8, 12) |
| **Female n (%)** | 0(0) | 1(100) | 0(0) | 44(51.2) | 75(50.3) | 90(55.2) | 14(58.3) | 25(47.2) | 29(50) | 58(52.7) | 101(49.8) | 119(53.8) |
| **Male n (%)** | 0(0) | 0(0) | 0(0) | 42(48.8) | 74(49.7) | 73(44.8) | 10(41.7) | 28(52.8) | 29(50) | 52(47.3) | 102(50.2) | 102(46.2) |
| **Median weight (IQR)** | 0(0) | 31.8(31.8, 31.8) | 0(0) | 28.1(23.3, 37.4) | 27.9(23.2, 36.6) | 28.3(22.4, 36.95) | 27(20.75, 33.825) | 25(21, 30.5) | 24.75(20, 30.325) | 27.8(22.025, 37.275) | 27(22, 34.7) | 27.5(21.7, 35.1) |
| **Median heights (IQR)** | 0(0) | 136(136, 136) | 0(0) | 136.5(125.25, 152) | 137(125, 148) | 136(126, 149.5) | 131.5(120, 145.25) | 130(120, 135) | 127.5(119, 138.75) | 135.5(124.25, 149) | 134(125, 145.5) | 135(123, 147) |
| ***T. trichiura* light infection n (%)** | 0(0) | 1(100) | 0(0) | 81(94.2) | 137(91.9) | 152(93.3) | 20(83.3) | 45(84.9) | 44(75.9) | 101(91.8) | 183(90.1) | 196(88.7) |
| ***T. trichiura* moderate and heavy infection n (%)** | 0(0) | 0(0) | 0(0) | 5(5.8) | 12(8.1) | 11(6.7) | 4(16.7) | 8(15.1) | 14(24.1) | 9(8.2) | 20(9.9) | 25(11.3) |

# Figure S1: Performance of classification models in the training set


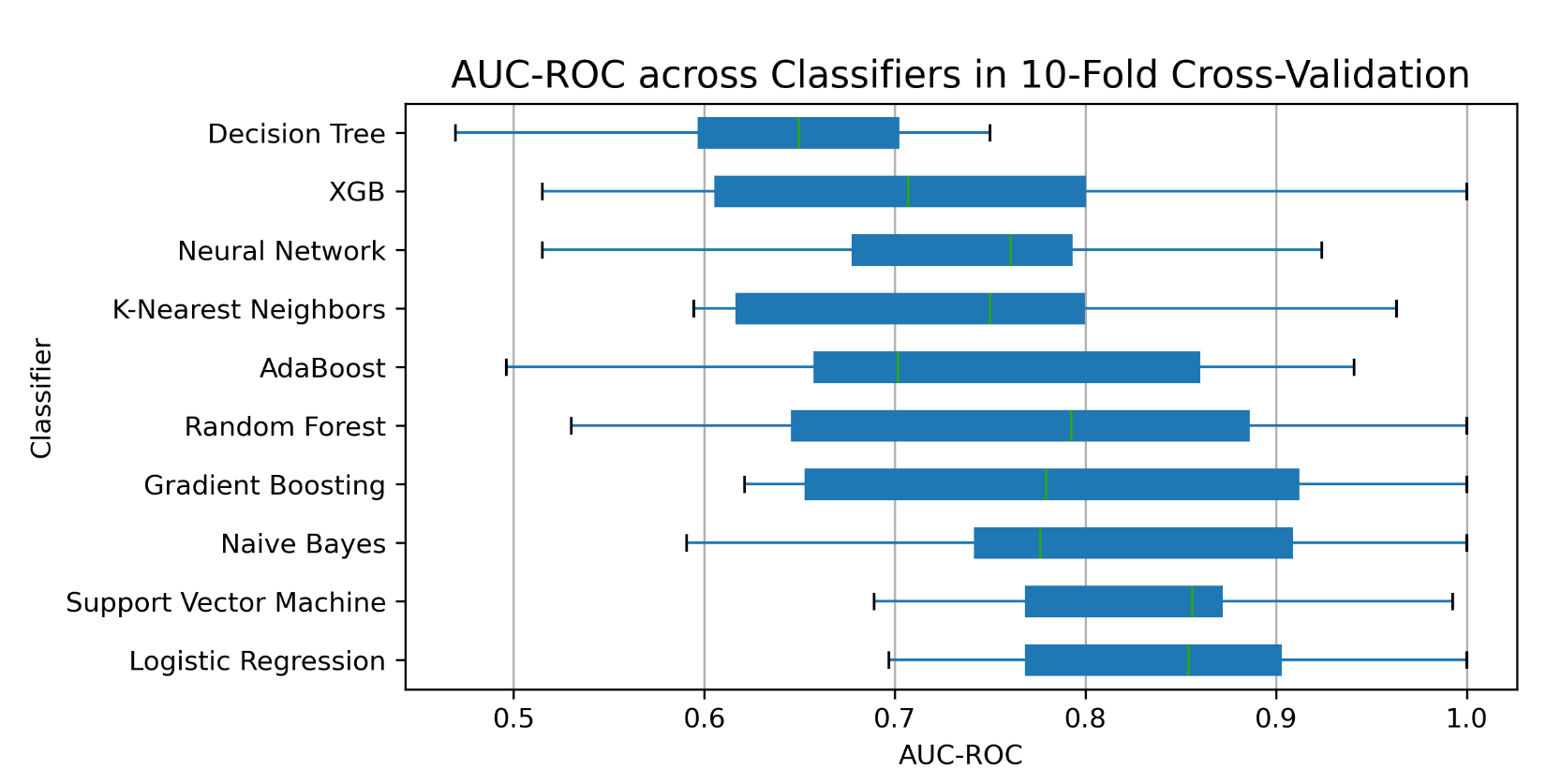

Supplement: Supplementary file 1 [file Table1.docx]
